# Book Notices

**Published:** 1889-03

**Authors:** 


### Book Notices.

A Reference Hand-book of Medical Sciences, embracing the entire range of Scientific and Practical Medicine, and allied Sciences, by various writers. Illustrated by chromo lithographs and fine wood engravings. Edited by Albert H. Buck, M. D., New York City. Vol. VII, New York, Wm. Wood & Co., 56 and 58 Lafayette Place, 1889. Quarto, cloth, pp. 795.

This volume brings the work down to Ter–Wor, alphabetically, and embraces many subjects of interest and importance. The chapter on Teratology, which embraces twenty-six pages, is most thorough and exhaustive. In it is given a history of every *lusns naturæ* in the human species that has ever been recorded, and most of them are illustrated with cuts representing the abnormality. It is unfortunate that the chapter was written before the remarkable case of the four-legged married woman described by Dr. Joseph Jones in the Transactions Louisiana State Medical Association was published; it is needed to make the record complete. This compilation of remarkable facts is alone worth the price of the work.

Volume VII contains also some very fine colored plates. It is uniform with the preceeding volumes, and is gotten up in good style.

---

The Pathology and Treatment of Displacements of the Uterus. By Dr. B. S. Schultze, professor of Gynecology in Jena. Translated from the German by Jameson J. Macan, M. A., M. R. C. S. Eng., and edited by Arthur V. Macan, M. B., M. Ch., Dublin. With 120 illustrations, New York, D. Appleton & Co., 1888. Cloth 8 vo. pp. 378, price $3.50.

One cannot fail to be impressed faborably with Dr. Schultze's book. He has gone deeply into the subject, and has strengthened the position taken by him some few years ago. Prof. Schultze has not much faith in efficacy of pessaries, He has found only two to fulfill the indications for displacements: his figure of eight and sleigh pessaries. He breaks up peritoneal adhesions bimannually, and claims that no bad results follow.

Of the Physician's Leisure Library Series, published by Geo. S. Davis, Detroit, Michigan, the following books have been received:

"Diseases of the Kidneys," by Dujardin Beaumetz, M. D., Paris, France. Translated from the fifth French edition by E. P. Hurd, M. D., Newberyport, Mass., 1888.

"Bright's Disease," by Alfred L. Loomis, M. D., New York.

"Hysteria and Epilepsy," by J. Leonard Corning, M. D. New York.

"Cliuical Lectures on Certain Diseases of the Nervous System," by Prof. J. M. Charcot, M. D., Paris, France. Translated by E. P. Hurd, M. D., Newberyport, Mass.

